# Supplementary figures and images for: Brown adipose tissue and skeletal muscle coordinately contribute to thermogenesis in mice
Source: eLife. 2025 Oct 27;13:RP99982. doi: 10.7554/eLife.99982 (PMC12558653; doi:10.7554/eLife.99982)

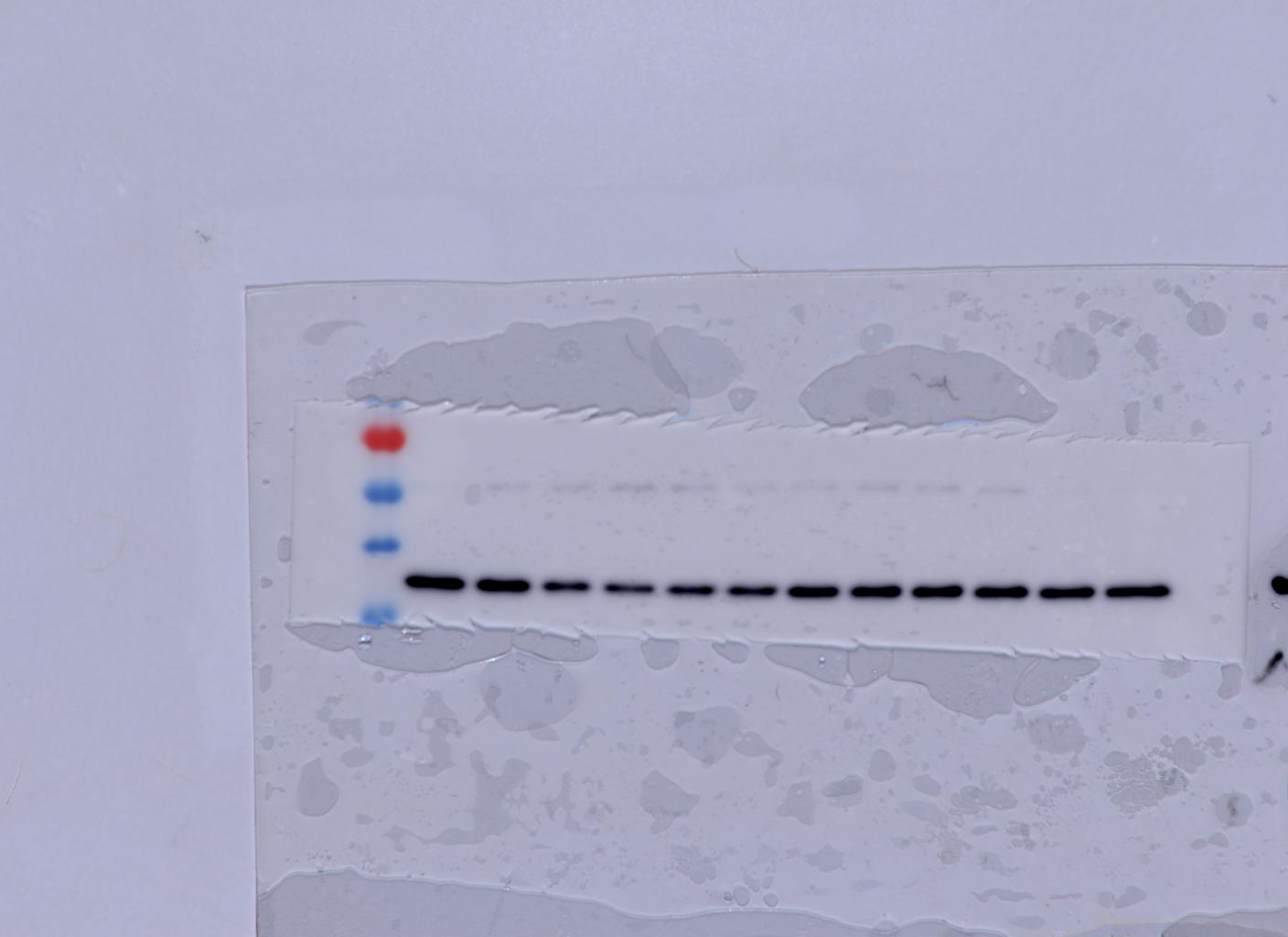

Supplement: Figure 1—source data 2. [file elife-99982-fig1-data2.zip › Figure 1E-F Raw WB data/PGC1a Raw data/GAPDH_Gel1.tiff]

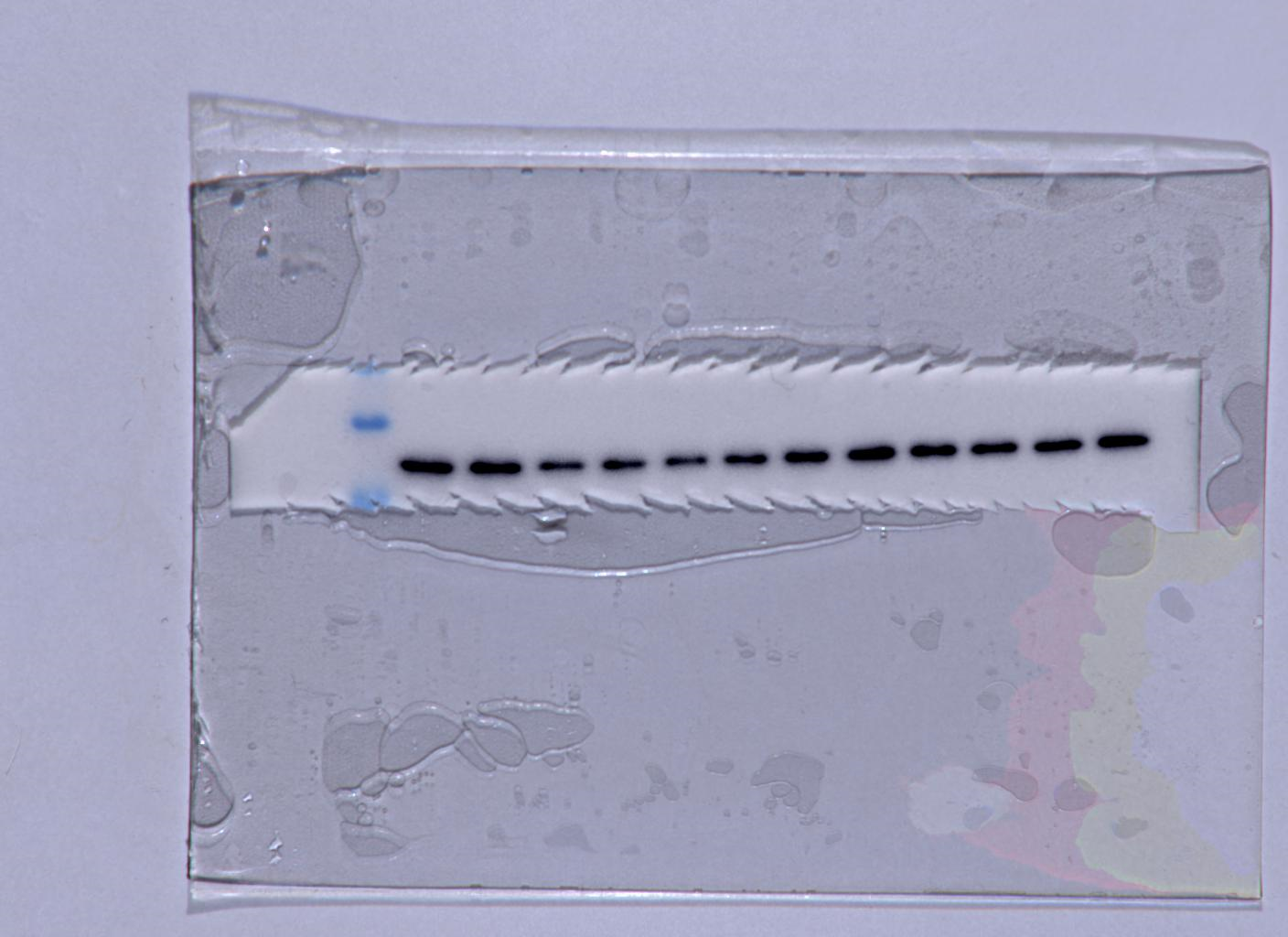

Supplement: Figure 1—source data 2. [file elife-99982-fig1-data2.zip › Figure 1E-F Raw WB data/PGC1a Raw data/GAPDH_Gel2.tiff]

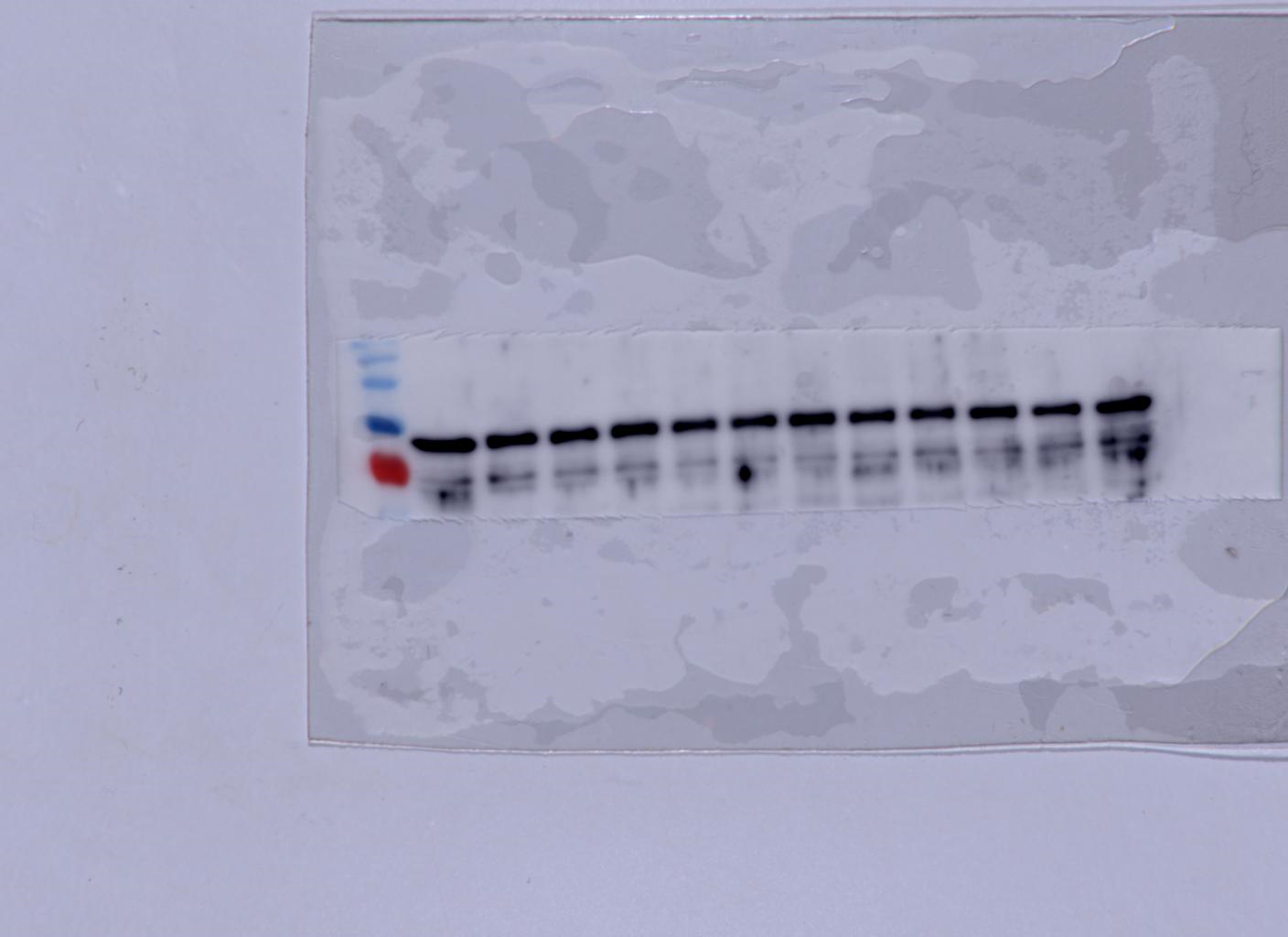

Supplement: Figure 1—source data 2. [file elife-99982-fig1-data2.zip › Figure 1E-F Raw WB data/PGC1a Raw data/PGC1a_Gel1.tiff]

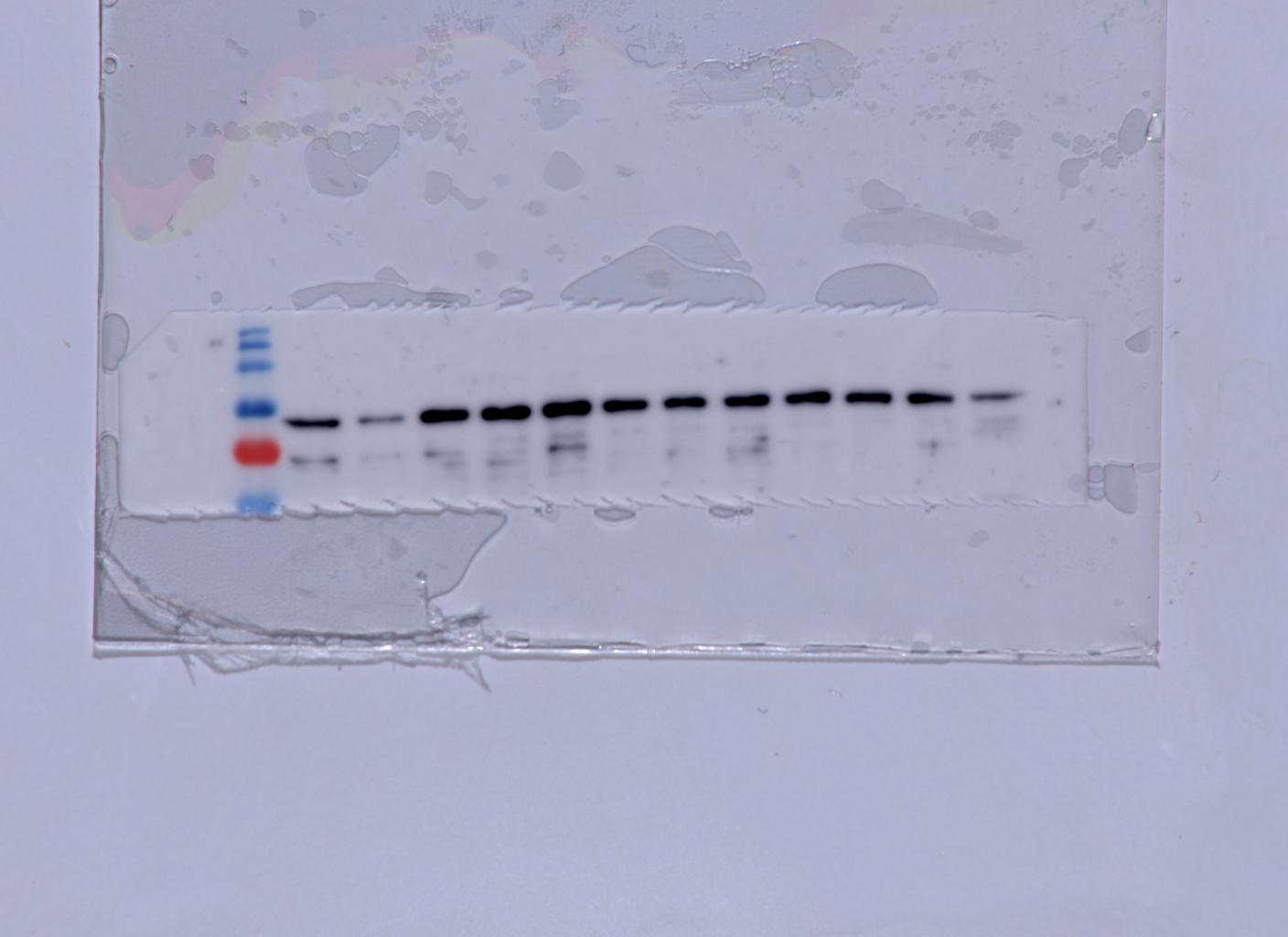

Supplement: Figure 1—source data 2. [file elife-99982-fig1-data2.zip › Figure 1E-F Raw WB data/PGC1a Raw data/PGC1a_Gel2.tiff]

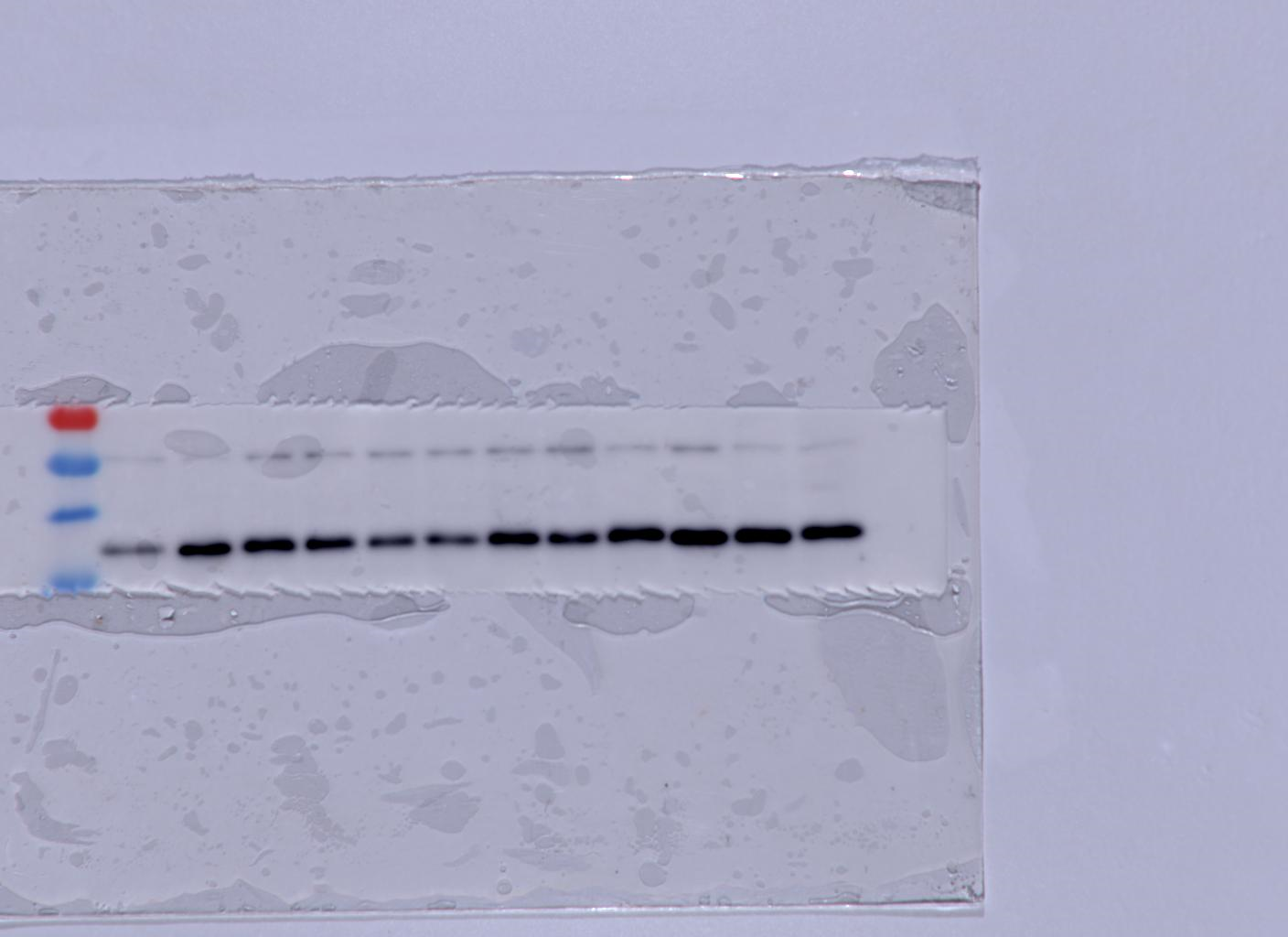

Supplement: Figure 1—source data 2. [file elife-99982-fig1-data2.zip › Figure 1E-F Raw WB data/UCP1 Raw data/GAPDH_Gel1.tiff]

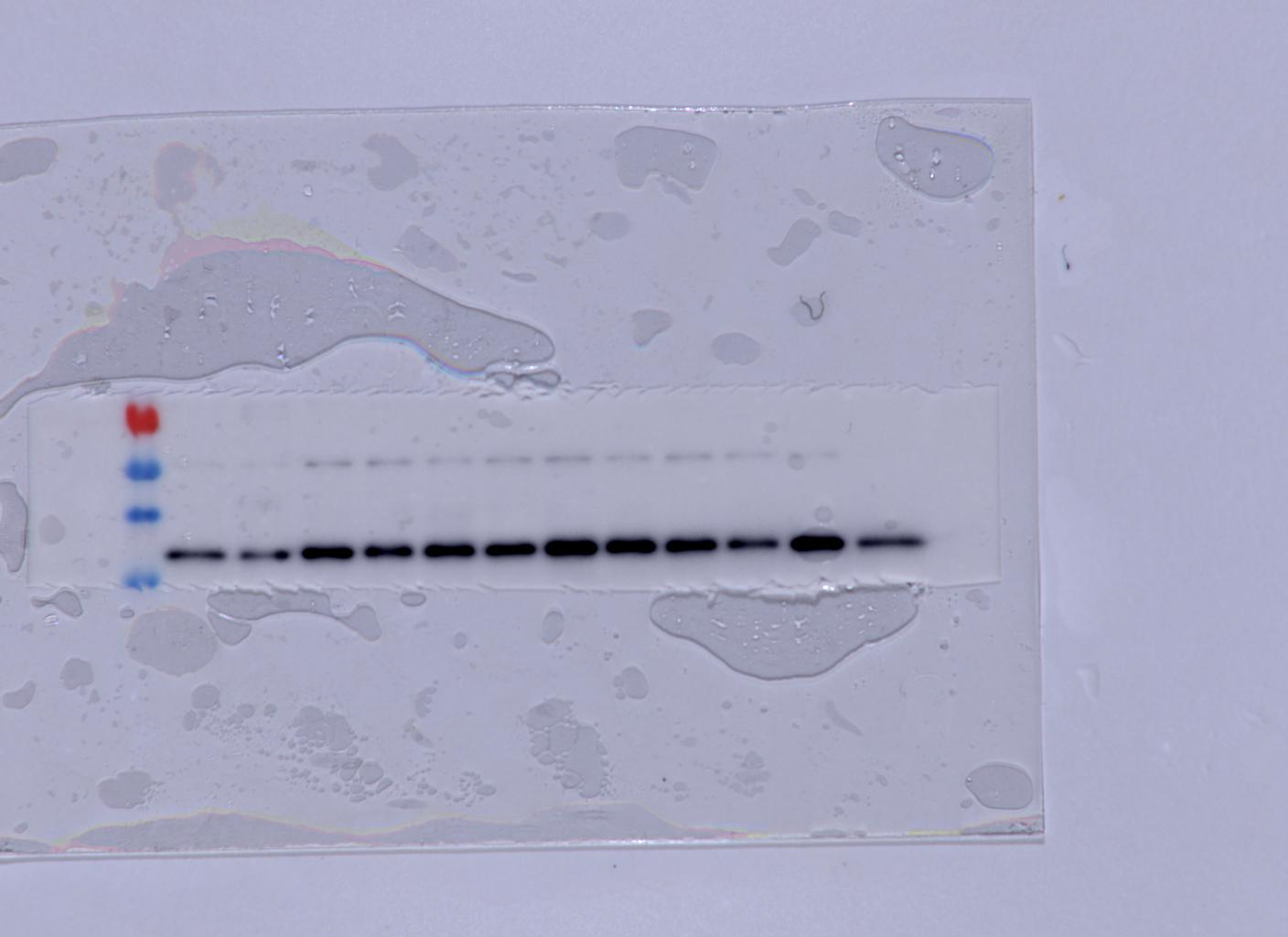

Supplement: Figure 1—source data 2. [file elife-99982-fig1-data2.zip › Figure 1E-F Raw WB data/UCP1 Raw data/GAPDH_Gel2.tiff]

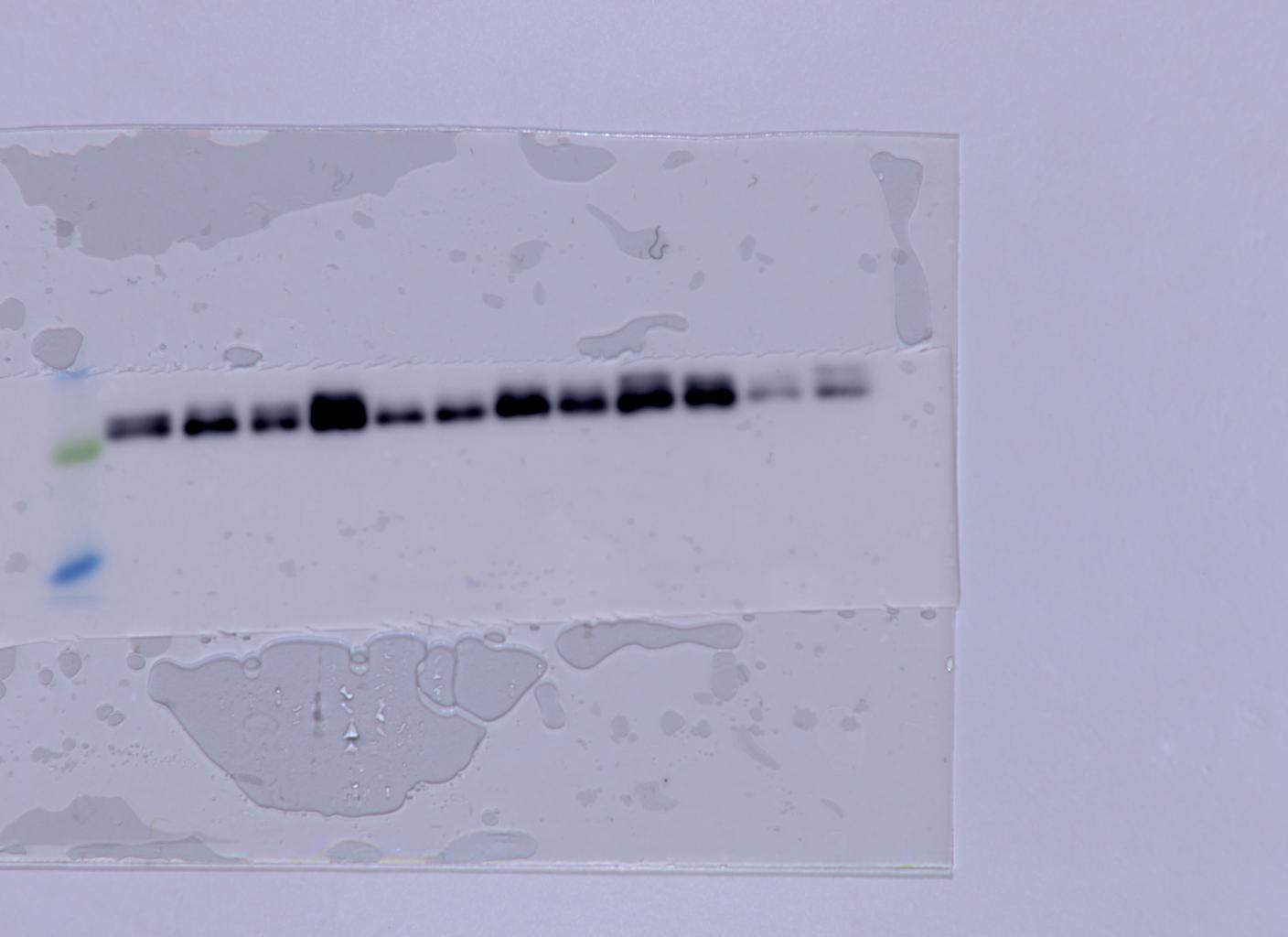

Supplement: Figure 1—source data 2. [file elife-99982-fig1-data2.zip › Figure 1E-F Raw WB data/UCP1 Raw data/UCP1_Gel1.tiff]

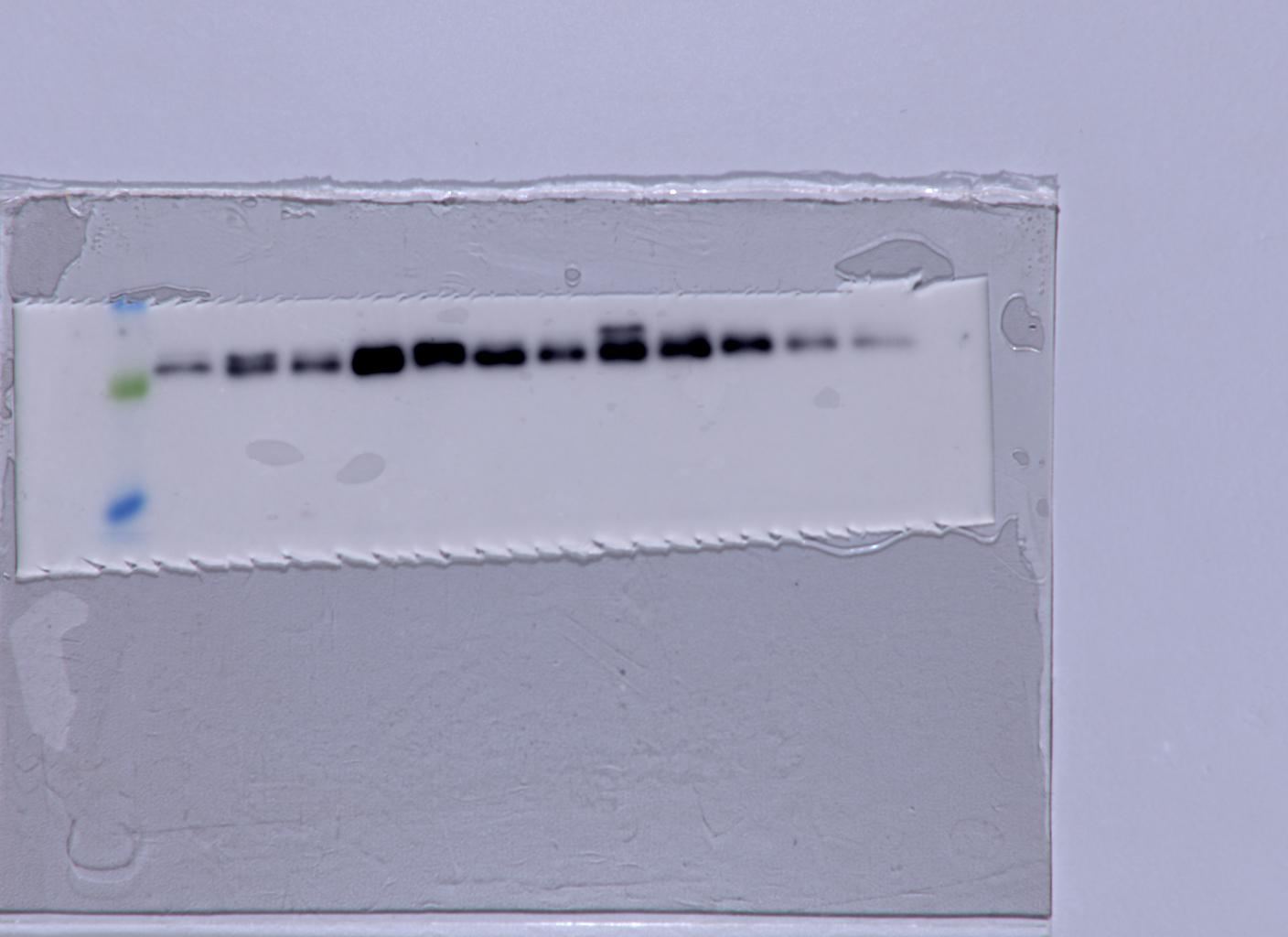

Supplement: Figure 1—source data 2. [file elife-99982-fig1-data2.zip › Figure 1E-F Raw WB data/UCP1 Raw data/UCP1_Gel2.tiff]

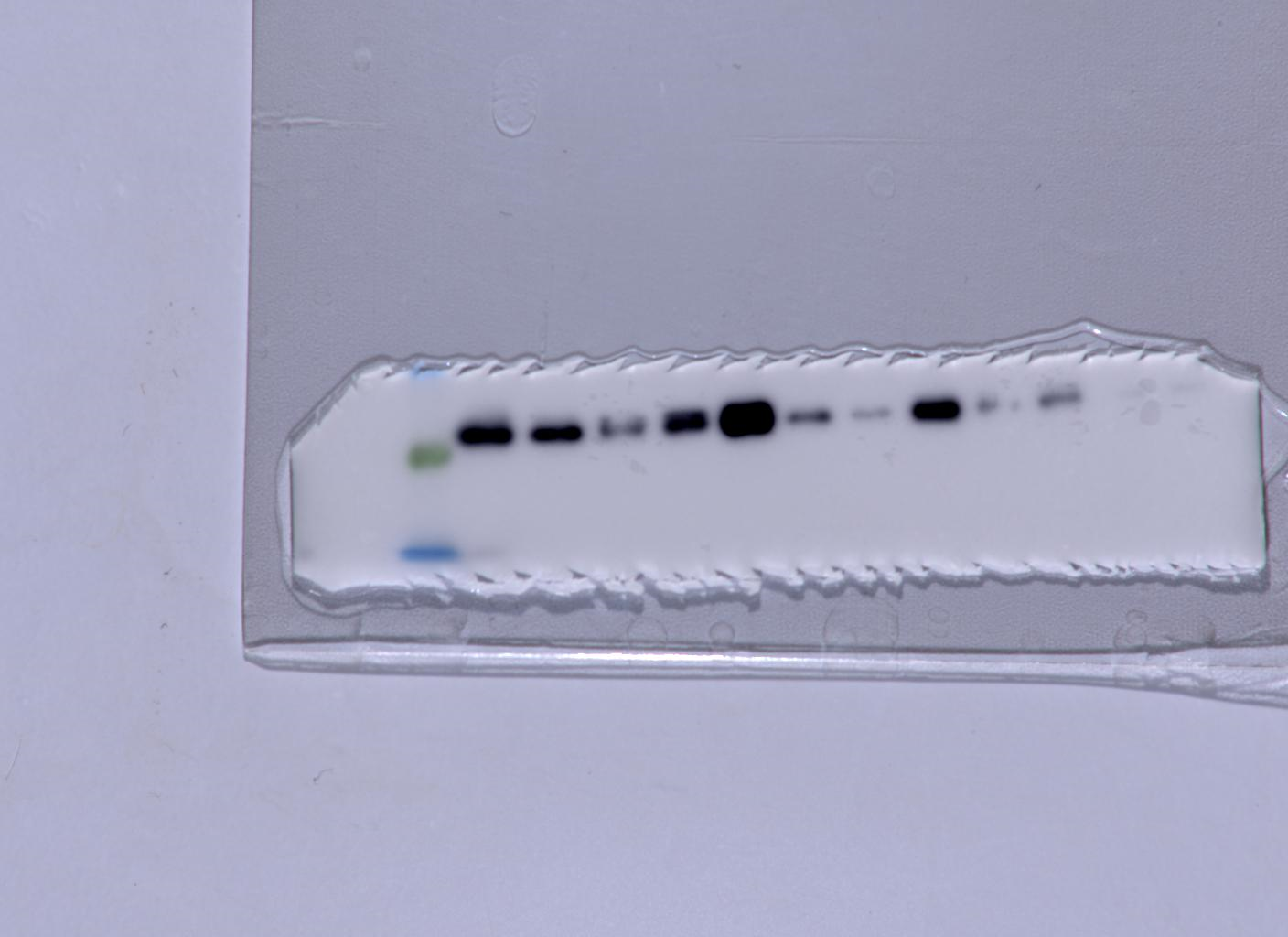

Supplement: Figure 1—source data 2. [file elife-99982-fig1-data2.zip › Figure 1E-F Raw WB data/UCP1 Raw data/UCP1_Gel3.tiff]

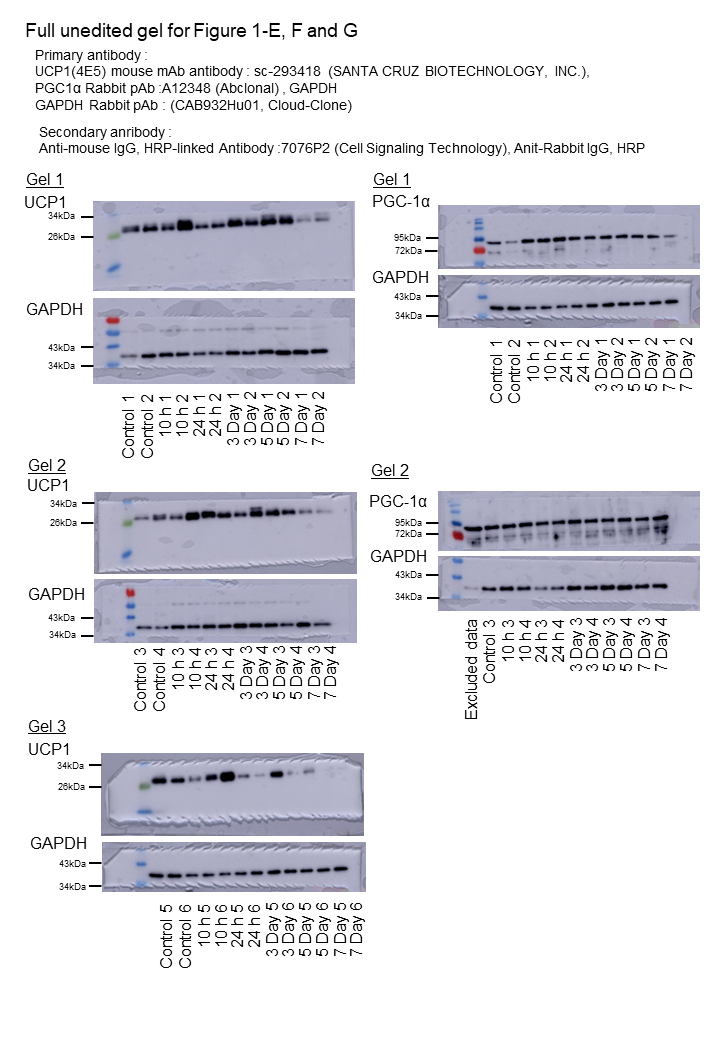

Supplement: Figure 1—source data 3. [file elife-99982-fig1-data3.zip › Labelled WB data/Source blot data for Figure 1E-G.TIF]
