# Supplementary material for: Brown adipose tissue and skeletal muscle coordinately contribute to thermogenesis in mice
Source: eLife. 2025 Oct 27;13:RP99982. doi: 10.7554/eLife.99982 (PMC12558653; doi:10.7554/eLife.99982)
Supplement: Supplementary file 1. [file elife-99982-supp1.pdf]

Supplemental information

Brown Adipose Tissue and Skeletal Muscle Coordinately Contribute to Thermogenesis in Mice

Yuna Izumi-Mishima, Rie Tsutsumi, Tetsuya Shiuchi, Saori Fujimoto, Momoka Taniguchi, Mizuki Sugiuchi, Manaka Tsutsumi, Yuko Okamatsu-Ogura, Takeshi Yoneshiro,Masashi Kuroda, Kazuhiro Nomura, and Hiroshi Sakaue

Supplementary File_Table 1A. Proportion of immobilized skeletal muscle weight for cast-immobilized mice.

Body weight and tissue weights for skeletal muscle of the posterior cervical region of 12-week-old male C57BL/6J mice are shown.

Mouse	Body weight (g)	Hind limbs (g)	Fore limbs (g)	Other (g)	Total (g)	Hind limbs (%)
1	27.93	1.762	0.454	1.256	3.472	50.74885
2	28.58	1.516	0.517	1.560	3.593	42.19315
3	30.06	1.958	0.476	2.256	4.690	41.74840
4	31.66	1.805	0.536	1.797	4.138	43.62011
Average	29.5575	1.76025	0.49575	1.71725	3.97325	44.57763

Supplementary File_Table 1B. Soleus and gastrocnemius tissue weights for individual mice subjected to iBAT removal (or sham surgery) followed by cast immobilization for up to 7 days.

Sham (mg)				Removed (mg)			
Group	No.	Soleus	Gastrocnemius	Group	No.	Soleus	Gastrocnemius
Control	1	23	333.4	Control	1	17.6	310.7
	2	20.4	299.1		2	17.4	271.1
	3	18.2	303.4		3	16.1	229.5
	4	17.9	308.4		4	18.4	287.8
	5	18.9	302		5	20.2	283.9
	6	17.6	289.5		6	14.5	304.5
	7	21.6	285.9		7	16.7	281.8
10H	1	22.7	316.3	10H	1	23.9	320
	2	17.9	284.8		2	18.9	309
	3	16.6	268.2		3	15.9	270.7
	4	15.3	253.6		4	16.3	268.8
	5	18.3	286.8		5	16.8	278.5
24H	1	21.2	296.1	24H	1	18.2	348.3
	2	18.5	296.5		2	13.1	277
	3	18.5	278.1		3	15.5	279.4
	4	18	273		4	16.6	276.9
	5	15.2	271.4		5	16.5	272.8
Day3	1	ND	321.8	Day3	1	18.8	372.4
	2	20.1	271.6		2	19.4	301
	3	14	277.7		3	19.1	291.1
	4	15.8	253.3		4	15.2	254.3
Day5	1	17.4	278.4		5	17.6	275.5
	2	14.1	259.5	Day5	1	16.2	286.4
	3	14.8	273.7		2	16.7	238.3
	4	14	200.8		3	14.9	278.5
	5	10.7	213.3		4	16.5	222.2
Day7	1	14.4	256.6		5	13.4	262.8
	2	15.4	252.7	Day7	1	19	287.8
	3	14.4	239.8		2	18	251.4
	4	14.8	261.3		3	16.7	235.8
	5	16	268.2		4	11	239.2
	6	15.6	264.5		5	12.7	234.4
					6	11.8	244.8

Supplementary File_Table 1C. Sequences (5′→3′) of PCR primers.

Transcript (Mouse)	Forward primer	Reverse primer
Gapdh	AAAATGGTGAAGGTCGGTGTG	TTGACTGTGCCGTTGAATTTG
Ucp1	CTCAGCCGGAGTTTCAGCTT	GTTTTTGCCAGGGTGGTGAT
Ucp2	TCTTGCCGATTGAAGGTCCC	CTAGCCCTTGACTCTCCCCT
Ucp3	TGTCTCTGCCTTTGGAGCTG	GGCCCTCTTCAGTTGCTCAT
Sln	AGGGGCCATGCTATACTCCA	TGGGCAGCCTACAAGAACAG
Camk2a	GGTCAGGAGTATGCTGCCAAG	CCCACCAGTAACCAGATCGAA
Fbxo32	AGGAGCGCCATGGATACTGT	GAAGTTCTTTTGGGCGATGC
Trim63	GACAGTCGCATTTCAAAGCA	AACGACCTCCAGACATGGAC
Ppargc1a	TCACACCAAACCCACAGAAA	TCTGGGGTCAGAGGAAGAGA
Tfam	CGGCTCAGGGAAAATTGAAG	AGCCATCTGCTCTTCCCAAG
Bcat2	TTCCAGAACCTCACGCTACAC	TAGCAGAACGTAGCATCCTGTC
Bckdha	AGGAGGTGCTGAAGTTCTACC	CGCCATAGTTGGTCATGTAGAAG
Slc1a5	GCAGTGCACCAACCAAAGAG	CCAGGCCCAGGATGTTCATT
Slc7a5	TTTTGCTCGGCTTCATCCAG	ACAACTTCTGCTGCAGGTTG
Slc38a2	CCTTCTGGTGTCCCTTGTCC	CTGCGGTGCTATTGAATGCC
Slc43a1	CCTGGGCCTCCTACTTCTCT	TGCAGGTAGAAAGCCACAGG
Slc25a10	CAGGATGCAGAACGACATGAA	ACCATCCAGGGCATGAGAGTA
Slc25a44	TCGCTGCTAACGTACATCCC	AGACAATGTGAGGGCACTCC
Cd36	GCCAAGCTATTGCGACATGA	AAAGGCATTGGCTGGAAGAA
G6pc1	AAGACTCCCAGGACTGGTTCATCC	TAGCAGGTAGAATCCAAGCGCG
Pck1	TGCTGATCCTGGGCATAACTAACC	TGGGTACTCCTTCTGGAGATTCCC
Il1b	TGGCAACTGTTCCTG	GGAAGCAGCCCTTCATCTTT
Il6	CACAGAAGGAGTGGCTAAGGACCA	ACGCACTAGGTTTGCCGAGTAGA
Il10	TTTGAATTCCCTGGGTGAGAA	GCTCCACTGCCTTGCTCTTATT
Il15	ACATGGCCCTCTGGCTCTT	AGCTGCCATCCATCCAGAA
Tnfa	TGAACTTCGGGGTGATCGGT	GTTTGCTACGACGTGGGCTAC
Mcp-1	CTGTTCACAGTTGCCGGCTG	AGCTTCTTTGGGACACCTGCT
Irisin	GAAGGAGATGGGGAGGAACC	GGTGTGCTGGTTTCTGATGC
Fgf21	GGATCGCCTCACTTTGATCC	ATCCTGGTTTGGGGAGTCCT
Saa3	ATGCTCGGGGGAACTATGAT	TCCATGTCCCGTGAACTTCT
Socs3	CTTTTCTTTGCCACCCACGG	CGACAAAGATGCTGGAGGGT
Crh	TCAGAGCCCAAGTACGTT	AGGGACTTCTCTCAGGAT
Bmp8b	CCTCGAACAGCAAGACCACT	GCACTCCCCAAGCACAGTAAT
